# Low Expression of MicroRNA335-5p Is Associated with Malignant Behavior of Gallbladder Cancer: A Clinicopathological Study

**DOI:** 10.31557/APJCP.2019.20.6.1895

**Published:** 2019

**Authors:** Naseem Fatima, Anand Narain Srivastava, Jaya Nigam, Syed Tasleem Raza, Saliha Rizvi, Zainab Siddiqui, Vijay Kumar

**Affiliations:** 1 *Department of Pathology, *; 2 *Department of Biochemistry, Era’s Lucknow Medical College and Hospital, *; 2 *Department of Surgical Gastroenterology, *; 4 *Department of Surgical Oncology, King George’s Medical University, Lucknow, India. *

**Keywords:** miRNA 335-5p, real-time PCR, Gallbladder inflammatory diseases, Gallbladder cancer, prognosis

## Abstract

**Background:**

MicroRNAs (miRNAs) are non-coding RNAs that regulate multiple cellular processes during cancer progression, identified to be involved in tumorgenesis of several cancers including cancers of digestive system. However its role in gallbladder inflammatory disease (GID) and gallbladder cancer (GBC) has not been well documented. The present study was aimed to investigate the clinical significance of hsa-miRNA-335-5p (miR-335) in GBC and GID.

**Subjects and Methods:**

This prospective case control study, conducted from July 1, 2014 to December 1, 2017 in Era’s Lucknow Medical College & Hospital, India, evaluated miR-335 expression by real-time polymerase chain reaction. Hundred tissue samples GID (control; n=50) and GBC (case; n=50) were studied. Relative quantification of target miR-335 expression was examined using the comparative cycle threshold method. Their expression was correlated with different clinicopathological parameters. Fishers’ exact test, Student’s t-test, and Chi-square test were used as appropriate for data analysis. Kaplan-Meier methods were used to calculate overall and disease-free survival rate. Two sided P<0.05 was considered as significant.

**Results:**

miR-335 expression was found to be significantly low in GBC lesions when compared with GID lesions (P<0.001). The low expression level of miR-335 was correlated with histological grade (P=0.007), clinical stage (P<0.001), lymph node metastasis (P<0.001) and liver metastasis (P=0.016). Reduced expression of miRNA-335 was associated with a shorter median overall survival (7 months vs. 25 months) in GBC patients (P<0.001).

**Conclusions:**

Down regulation of miR-335 is associated with the severity of the disease and thus indicate that miR-335 expression may serve as prognostic marker for GBC.

## Introduction

Gallbladder cancer (GBC) is highly aggressive and incurable disease representing the most common malignancy of the biliary tract with three-fold higher incidence in females (Varshney et al., 2002; Mekeel et al., 2007). GBC shows striking geographical predictions in its incidence with the highest figures reported in India and Chile and relatively low levels in many western countries (Hundal et al., 2014). The overall 5 years survival of less than 5% has been reported (Goetze et al., 2010). The poor outcome for GBC is ascribed to late presentation, absence of specific symptoms, rapid and silent progression, ineffective therapy and limited knowledge of its etiology (Kanthan et al., 2015). To date, GBC continues to be a challenge with regards to its early stage diagnosis and optimal management of advance disease, and requires evaluation of all factors including molecular markers associated with the disease. There are many reports focused on the genetic and epigenetic alterations in GBC, which involve modifications in the expression of tumor suppressor genes and oncogenes. However, there are very few studies focused on miRNA-based epigenetic modifications involved in gallbladder carcinogenesis.

MicroRNAs (miRNAs) are small, non-coding ribonucleic acids (RNAs). MiRNAs play a role on various biological processes such as differentiation, proliferation and apoptosis and control multiple gene involved in cancer. Accumulating studies indicate that miRNAs display aberrant expression patterns and functional abnormalities in many types of cancers. They act either as tumor suppressors or oncogenes according to the roles of their target genes (Davis et al., 2010; Peng et al., 2016; Stanczyk et al., 2008; Berezikov et al., 2005). Changes in their expression can be used as robust and important biomarkers for cancer risk, diagnosis, prognosis, and can even be used as miRNA-based therapeutic targets. 

MiR-335 has been reported to differentially express in benign and malignant tumors (Schmitz et al., 2011). It is known to be associated with increased risk and prognosis of various cancers (He et al., 2016). MiR-335 has been found deregulated in breast cancer cells, gastric cancer cells, and ovarian cancer cells (Tavazoie et al., 2008; Sandoval et al., 2017; Cao et al., 2014). But its association with aggressive behavior of GBC and gallbladder inflammatory disease (GID) has not been well evaluated. It was hypothesized that miR-335 expression would be highly down regulated in advanced stage of GBC, when compared primary stage of GBC, thus it might work as molecular marker for disease progression. Therefore we investigated the role of miR-335 expression in tissue sample of patients with different stage of GBC and compared with tissue sample of patients with GID.

## Materials and Methods

*Patients*

This observational prospective case control study includes 50 cases of GBC who, were operated in the Department of Surgical Oncology, King George’s Medical University, Lucknow and Department of Surgery, Era’s Lucknow Medical College and Hospital, Lucknow, from July 2014 to December 2017. All patients suspected of having GBC based on clinical evaluation (Ultrasonography and computed tomography), who underwent for exploration for primary gallbladder cancer followed by either radial surgery or biopsy of metastatic lesion found during exploration. The control group comprised 50 patients of GID cases of chronic calculus cholecystitis and adenomatous hyperplasia without any gallbladder polyp or adenoma disease based on clinical evaluation and confirmed by ultrasound examination and underwent laparoscopic or open Cholecystectomy were included in the study.

*Ethical Compliance*

Protocol and procedures employed were reviewed and approved by the Institutional Ethics Committee of Era’s Lucknow Medical College and Hospital, Lucknow India, and was in accordance with Helsinki declaration (ELMC/R_Cell/EC/2014/01, July 11, 2014). 

The patients enrolled in the study provided a written informed consent for participation in this research as well as for presentation of data. The exclusion criteria were immunodeficiency disorder, patients with other cancer or who received prior chemo-radiotherapy or operated earlier. 

Tissue samples were taken into two portions; one portion of the tissue was collected in 10% buffered formalin and embedded in paraffin for histopathological examination and pathological staging was done according to American Joint Committee on cancer (AJCC) tumor node metastasis (TNM) staging 7^th^ edition 2010 (Edge et al., 2010). The other portion was collected in RNA later (Sigma-Aldrich ), kept at -80°C till further miR-335 processing. 

*Methods*

Total ribonucleic acid (RNA) was extracted from tissue by using the mirVANA miRNA isolation Kit according to the manufacturer’s protocol. The RNA concentration was determined by Nanodrop 2000 spectrophotometer (Thermo Fisher Scientific) and sample was stored at -20°C. The miR-335 and RNU6B (internal reference gene)-specific complementary de-oxyribonucleic acid (cDNA) were reverse-transcribed from two micrograms total RNA using TaqMan MicroRNA Reverse Transcription kit, Applied Biosystems, (USA). TaqMan Universal PCR Master Mix, No AmpErase (Uracil-N glycoslyase). Individual RT-PCR assays were performed in a 20μL reaction volume on a RT- PCR system (Applied Biosystems StepOnePlus™) to detect miR-335 expression in tissue. The thermal cycling conditions consists of optional AmpErase UNG activity hold at 50°C for 2 minutes, enzyme activation hold at 95°C for 10 minutes, further 40 PCR cycles of denaturation at 95°C for 15 seconds and annealing and extension step at 60°C for 60 seconds. The cycle threshold (Ct) value was recorded for both miR-335 and RNU6B, and the relative quantification of target miRNA expression was expressed as (2^-ΔΔCt^), where ΔCt =Ct miR^-^335-Ct RNU6B. The correlation was done in between Ct of target miRNA with respect to RNU6B. Each sample was examined in triplicate. 

*Statistical analysis*

The sample size was calculated using the formula n= Zα^2^pq/ L^2^, where (p=57.83% , q=100-p, Type I error α =5%, Allowdance error L = ¼ th of p, Power of study = 75% , Data loss = 10%) (Reference percentage reduced expression of miR-335) final size of the sample is 50 in each group.

All the clinical details and the experimental data were analyzed using GraphPad Prism version 5.0 by GraphPad Software, Inc. California, USA. Descriptive statistics of study subjects are either represented as mean with standard deviation or frequency with percentages. Normality of the data distribution was determined by Smirnov- Kolmogorov test. The chi-square (χ^2^) test was used to test for a statistically significant relationship between two categorical study variables. The association between miR-335 expressions and clinopathological characteristic of gallbladder cancer patients was investigated by using Chi-square test or Fisher exact test. The cut-off values of miR-335 was set by the median value of the expression levels, and were divided into two groups according to high and low expression levels of miR-335. Correlation analysis was performed using Pearson correlation method. Survival curves were plotted using the Kaplan-Meier method and compared using the log-rank test. The Cox proportional hazards model was used to analyze the significance of various parameters for survival. Two tailed P value <0.05 was considered statistically significant. 

## Results

**Figure 1:**
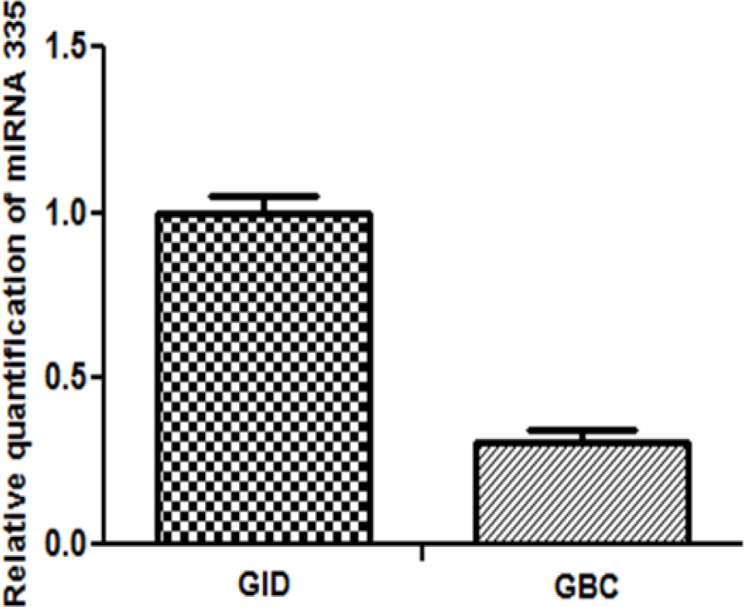
Low Expression Level of miRNA-335-5p in 50 Cases of Gallbladder Cancer (GBC) and 50 Cases of Gallbladder Inflammatory Disease Tissue (GID)

A total of 100 patients (GBC (n=50; GID (n=50)) were evaluated for the expression of miRNA - 335. Study subjects were predominantly female. Average aged subjects 50.52±1.43 in GBC and 40.14±1.35 in GID cases; with high prevalence of patients with gallstones in both case and control group (Table 1). Quantitative RT-PCR analysis showed significant decrease in the expression of miR-335 in GBC as compared to GID (P<0.001) (Figure 1).

In this study the median expression level of miR-335 (0.223) was used as a cutoff point to divide all 50 GBC patients into two groups. GBC patients who expressed miR-335 with values less than the median expression level were assigned at low expression group (mean expression value =0.093; n=25), and those with expression above the cutoff value were assigned to the high expression group (mean expression value =0.530; n=25).

Significant negative correlation was found between the low expression of miR-335 with advanced clinical stage (P<0.001), tumor differentiation (P=0.007), lymph node metastasis (P<0.001) and liver metastasis (P=0.016). However miR-335 expression was not significantly related to sex, age, presence or absence of gallstone, or pain in abdomen (P>0.05) (Table 2).

**Figure 2:**
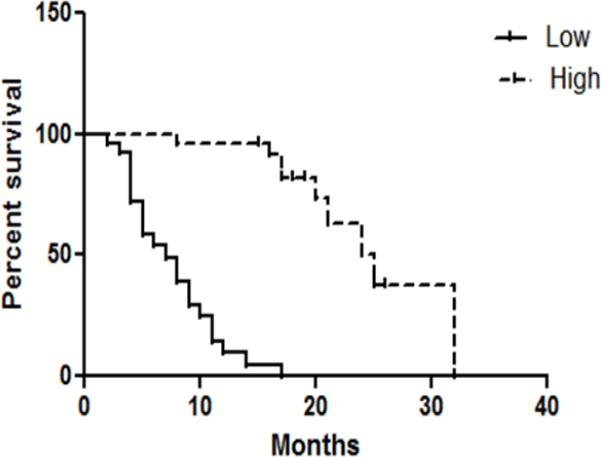
Kaplan-Meier Overall Survival Curve of GBC Patients (n=50) in Relation to Relative Quantification of miR-335 Expression. The survival of patients with low miR-335 expression was significantly poorer than those with high miR-335 expression (7 months vs. 25 months) (P<0.001).

The association between median overall survivals of GBC patients was investigated by Kaplan-Meier method and log-rank test (Figure 2). GBC patients with low miR-335 expression tend to have shorter median survival than those with high miR-335 expression. (7 months vs 25 months) (Hazard Ratio= 0.28, 95% CI: 0.1805 to 0.7405, P<0.001)

**Table 1:** Characteristics of Gallbladder Cancer (GBC) and Gallbladder Inflammatory Disease (GID) Patients

Characteristics	Control (GID) (n=50)	Cases (GBC) (n=50)
Age (mean±S.E) (Range) (Years)	40.14±1.35 (20-55)	50.52±1.43 (32-70)
Gender:		
Male	14 (28%)	8 (16%)
Female	36 (72%)	42 (84%)
Gall stones:		
Present	42 (84%)	31 (62%)
Absent	6 (12%)	19 (38%)
Pain in upper abdomen		
Present	All	44 (88%)
Absent	0	6 (12%)
Tumor differentiation		
Well differentiated	-	31 (62%)
Moderately differentiated	-	12 (24%)
Poorly differentiated	-	7 (14%)
Staging		
I-II	-	23 (46%)
III-IV	-	27 (54%)
Lymph node metastasis		
Yes	-	34 (68%)
No	-	16 (32%)
Liver Metastasis		
Yes	-	7 (14%)
No	-	43 (86%)

Data are presented as n (%)

**Table 2:** Correlation of miRNA-335-5p Expression with Various Clinicopathological Factors for Gallbladder Cancer (GBC)

Clinopathological Factors	Total number of cases	miRNA-335 expression		P-Value
		High (n=25)	Low (n=25)	
Age (mean+ S.E)	50.52±1.43 (32-70)	Above 51 (16) Below 51 (9)	Above 51 (14) Below 51 (11)	0.773
	
Gender				
Male	8	6	2	
Female	42	19	23	0.246
Gall stone				
Present	31	15	16	
Absent	19	10	9	1.00
Pain in abdomen				
Present	44	21	23	
Absent	6	4	2	0.667
Tumor differentiation				
Well	28	19	9	0.007
Moderate	13	5	8	
Poor	9	1	8	
Staging				
I-II	23	20	3	
III-IV	27	5	22	<0.001
Lymph node metastasis				
Yes	34	10	3	<0.001
No	16	15	22	
Liver Metastasis				
Yes	10	1	9	
No	40	24	16	0.016

*P<0.05, statistically significant difference; (S.E:Standard error)

## Discussion

In this study, we observed that down expression of miR-335 was significantly correlated with higher stage of disease. Also decreased expression of miR-335 was significantly associated with tumor differentiation, lymph node metastasis, liver metastasis and correlates with shorter survival. Down-regulated expression of miR-335 was found higher in patients with stage (III-IV) of disease. In recent years, miRNAs have been found to play a role in multiple human diseases including cancers, because they have great power to impact gene expression through posttranscriptional way. Various kinds of miRNAs have been found to correlate with tumorigenesis or tumor progression. For example, miRNA 21 was up-regulated in glioma compared with normal brain and related to cell apoptosis, tumor proliferation, invasiveness, and migration (Chai et al., 2018). Furthermore, many miRNAs have been found to correlate to tumor prognosis in GBC such as miRNA 34, miRNA 145, miRNA 146b, and miRNA 218 (Jin et al., 2014; Leteliar et al., 2014; Lv et al., 2017; Wang et al., 2017). 

As a potential tumor related miRNA, the gene locus of miR-335 is on chromosome 7q32.2 (Rojas et al., 2015). It has been demonstrated to function as an oncogenic or a tumor-suppressor miRNA in various human malignancies. MiR-335 is shown to be upregulated in myeloma, gliomas, and meningiomas, (Ronchetti et al., 2008; Shu et al., 2012; Shi et al., 2012) but downregulated in pancreatic adenocarcinoma, breast cancer and hepatocellular carcinoma (Gao et al., 2014; Dong et al., 2018; Cui et al., 2015). There have been controversial reports on the expression patterns of miR-335 even in the same cancer type. For example, Yan et al., (2012) reported that high frequency of recurrence and poor survival was associated with upregulated expression of miR-335 in gastric cancer. In contrast, Xu et al., (2012) found down regulation of miR-335 in gastric cancer, they reported that lymph node metastasis, poor pT stage, poor pN stage, and invasion of lymphatic vessels, was significantly associated with low expression of miR-335, suggesting a metastasis suppressor function of miR-335 in this disease. miR-335 has been demonstrated to play important roles gastrointestinal tract cancer including GBC (Li et al., 2017; Zhang et al., 2018; Wang et al., 2017; Feng et al., 2018; Lu et al., 2016; Zhang et al., 2014). However there has been scarcity of studies on GBC showing the prognostic and therapeutic importance of miR-335. 

In our study we observed lower expression of miR-335 in malignant lesions as compared to benign lesions of gallbladder. Similar observation was made by (Jiang et al., 2012; Sun et al., 2014 and Cao et al., 2014) in gliomas, colorectal cancer and ovarian cancer. 

Peng et al., (2013), reported down regulation of miR-335 in GBC and associated with aggressive tumor behavior. In line with these finding we also observed lower expression of miR-335 in GBC and found to be significantly associated with progression of the disease. 

Showing that down- regulation of miR-335 might play a crucial role in gallbladder cancer progression. 

In the present study, we found that miR-335 is an independent prognostic factor for patients with GBC, and patients with low miR-335 expression have poorer prognosis than those with high miR-335 expression. In fact, a recent study by Peng et al., (2013) on GBC had similar findings. Indicating that miR-335 expression may be a useful marker to predict patient survival in GBC [38]

However in contrast, Jiang et al., (2012) found that glioma patients with high miR-335 expression tumors had significantly shorter survival times than those with low miR-335 expression tumors. Although confirmation requires larger and more long-term studies, the variable interpretations of the significance of miR-335 expression between GBC and other types of cancers suggest that the actions and roles of miR-335 may depend on the cell type of tumor origin. 

Further investigations with target genes involved in miR-335 expression would help us better understand the clinical importance of miR-335 in cancer progression. Moreover it may also help us in discovering some novel therapeutic and prognostic possibilities for treating this dreaded disease.

Our study indicated that miR-335 expression was decreased in GBC and was associated with tumor progression. Furthermore, miR-335 expression was found to be a marker for predicting the clinical outcome of the GBC patients. Indicating that loss of miR-335 expression may be a useful prognostic marker and a therapeutic target for GBC.

## Declaration of Interest Statement

The authors report no declaration of interest.
